# Clonal spread of *mcr-1* in PMQR-carrying ST34 *Salmonella* isolates from animals in China

**DOI:** 10.1038/srep38511

**Published:** 2016-12-05

**Authors:** Xing-Ping Li, Liang-Xing Fang, Jia-Qi Song, Jing Xia, Wei Huo, Jin-Tao Fang, Xiao-Ping Liao, Ya-Hong Liu, Youjun Feng, Jian Sun

**Affiliations:** 1 National Risk Assessment Laboratory for Antimicrobial Resistance of Animal Original Bacteria, South China Agricultural University, Guangzhou, P. R. China; 2 Guangdong Provincial Key Laboratory of Veterinary Pharmaceutics Development and Safety Evaluation, South China Agricultural University, Guangzhou, P. R. China; 3 Department of Medical Microbiology and Parasitology, Zhejiang University School of Medicine, Zhejiang, P. R. China

## Abstract

Since initial identification in China, the widespread geographical occurrence of plasmid-mediated colistin resistance gene *mcr-1* in *Enterobacteriaceae* has been of great concern. In this study, a total of 22 *Salmonella enterica* were resistant to colistin, while only five isolates which belonged to ST34 *Salmonella enterica* serovar Typhimurium (*S.* Typhimurium) were *mcr-1* positive. Four of them shared nearly identical PFGE type, although they were from different host species and diverse geographical locations. All the *mcr-1*-positive *S.* Typhimurium exhibited multi-resistant phenotypes including ampicillin, streptomycin, gentamicin, florfenicol, nalidixic acid, tetracycline, trimethoprim-sulfamethox, in addition to colistin. The *oqxAB* and *aac*(*6*′)*-Ib-cr* genes were present alone or in combination in four (80.0%) and five (100%) isolates, respectively. The *mcr-1* gene was located on a transferable IncI2 plasmid in the four genetically related strains. In the other one strain, *mcr-1* was located on an approximately 190 kb IncHI2 plasmid. In conclusion, we report five *mcr-1-*positive *S.* Typhimurium/ST34 isolates. Both clonal expansion and horizontal transmission of IncI2-type plasmids were involved in the spread of the *mcr-1* gene in *Salmonella enterica* from food-producing animals in China. There is a great need to monitor the potential dissemination of the *mcr-1* gene.

Salmonellosis is one of important global public health zoonoses, causing life-threatening infections. Each year, there are an estimated 1.0 million *Salmonella* infections in the United States^1^. By contrast, 30 millions of infections every year in China, approximately 75% of the food-borne diseases, are attributed to this bacterium^2^. Unpublished data from the China CDC surveillance system indicated that the carriage rate of human salmonellosis is 549 per 100,000 people in 2013, which is higher than that in the USA in 2012 (16.4 per 100,000)^3^. *Salmonella enterica*, especially non-typhoidal *Salmonella* (NTS), is a leading cause of food-borne disease of humans and livestock worldwide^3^,^4^. Various animal species, such as poultry, pigs, cattle, and reptiles, are reservoirs for NTS. Human NTS infections are frequently due to the consumption of contaminated cooked or raw meat, milk, eggs, seafood, and other fresh products derived from animals^5^.

In recently year, emerging fluoroquinolone resistance prevalence has been identified in several *Salmonella* serovars and the resistance rate to fluoroquinolone has increased dramatically both in clinical and food-borne *Salmonella* isolates around the world^6^. Resistance toward quinolone and fluoroquinolone antimicrobials is mainly attributed to mutations of quinolone resistance-determining regions (QRDRs) and plasmid-mediated quinolone resistance (PMQR) mechanism including Qnr peptides (QnrA, QnrB, QnrS, QnrD and QnrC), AAC(6′)-Ib-cr and the efflux pumps QepA and OqxAB^7^. In our previous study, we have characterized a high prevalence of *oqxAB* (31.7%) in *Salmonella enterica* serotype Typhimurium (*S.* Typhimurium) isolated from food-producing animals in China. The *oqxAB* gene was present alone or in combination with other PMQR genes such as *aac*(*6*′)-*Ib-cr* and *qnrS1* genes. Interestingly, the *S.* Typhimurium isolates carrying *oqxAB* were clonally related as determined by PFGE and also defined as ST34 by MLST type^7^. A high prevalence of *oqxAB*-positive *S.* Typhimurium/ST34 was also detected in human clinical and food samples in Hong Kong in the same period^8^. It aroused a possibility that the *oqxAB*-positive S. Typhimurium/ST34 transmitted from food animals to humans via food chain. More recently, *Salmonella* species have been isolated that carried PMQR, can occur as a multiple drug resistance phenotype, which has caused international concern because it brought an even greater challenge for clinical treatment^9^,^10^.

Colistin (polymyxin E) is a cationic, multi-component, lipopeptide antibacterial agent discovered in the 1940s with significant activity against Gram-negative bacteria. Colistin has been used both in human and veterinary medicine for more than 50 years, although their parenteral usage in humans has been limited because of concerns about nephrotoxicity and neurotoxicity^11^. However, with a global increase in Gram-negative bacteria that are multidrug-resistant (MDR), extensively drug-resistant (XDR) and pandrug-resistant (PDR), colistin has been re-introduced as a last-resort drug for infections with such bacteria, which are frequently the cause of healthcare-associated infections^12^,^13^. In veterinary medicine, colistin is more widely used, mainly to treat Gram-negative infections of the intestinal tract^14^. In addition, colistin is used as a growth promoter in some countries duo to its great growth performance in pig and poultry production^14^,^15^.

Resistance to colistin in Gram-negative bacteria has been characterized by chromosomal mutations and was generally thought non-transferable by mobile genetic elements^11^. Specific regions mutations like *pmrAB* and *phoPQ*, which were related to structural changes (ParR-ParS two-component system) of the LPS at both the cytosol and periplasmic site of the cell membrane, could decrease colistin activity in *Klebsiella, E. coli* and *Salmonella enterica*^14^.

Recently, a plasmid-mediated colistin resistance gene (*mcr-1*) was firstly reported in food animals, food and humans in China^15^. The *mcr-1* gene was proved subsequently to be disseminated worldwide, mainly found in *E. coli, K. pneumonia and Salmonella spp*^15^,^16^. In China, *mcr-1* was dominantly identified in *E. coli, K. pneumonia, Enterobacter aerogenes*, and *Enterobacter cloacae* in many regions (Fig. 1), mainly in Guangdong^15^,^17^,^18^, Shanghai^15^, Zhejiang, Hubei, Jiangsu^19^, Sichuan^20^, Shandong, Anhui^21^, Chongqing^22^, Hong Kong^23^ and Taiwan^24^, whereas data on the transmission of *mcr-1*-mediated colistin resistance in *Salmonella spp.* are lacking. Although, during the preparation of this study, the prevalence of *mcr-1* among ESBL-positive *Salmonella spp.* isolates was investigated, but the presence of this gene in particular successful resistant clone has not been demonstrated^25^. In this study, we did a retrospective study to examine the emergence of the *mcr-1* gene in *Salmonella enterica* isolates from food-producing animals during 2007 to 2015.

## Results

### Antimicrobial susceptibility and detection of resistance genes

These isolates showed the minimal inhibitory concentrations (MIC) for colistin of 0.25 to 16 mg/L. A total of 22 *Salmonella* isolates were resistant to colistin with MIC ≥ 8 mg/L. The *mcr-1* gene was detected in only five colistin-resistant *S.* Typhimurium isolates with diverse origins (Table 1). Susceptibility testing showed that all the *mcr-1*-positive isolates in this study were resistant to nalidixic acid, olaquindox, ampicillin, streptomycin, gentamicin, florfenicol, tetracycline and trimethoprim-sulfamethox. All the five *mcr-1-*containing strains carried *aac*(*6*′)*-Ib-cr*, and four of them also carried *oqxAB* and *floR* (Table 1). Interestingly, all strains showed increased MIC values and exhibited intermediate resistance phenotype (strain GDS 79, GDS82, and GDS141) or resistance phenotype (strain S01) but except strain GDS78 retained sensitivity to ciprofloxacin according to the cutoff of CLSI, although most of them harboured one or more PMQR genes (Table 1).

### Transfer of *mcr-1* gene

Four transconjugants were successfully obtained from the five *mcr-1*-positive isolates. Conjugation and transformation tests were not successful for strain S01 despite repeated attempts. All transconjugants showed 32-fold increase in the MICs of colistin, in comparison with the recipient *E. coli* C600 (0.125 mg/L). However, other antibiotic-resistant phenotypes could not be co-transfered with colistin except for strain GDS78. The transconjugant of GDS78 was multidrug-resistant and showed resistance to more than five antibiotics, in addition to colistin (Table 1).

### Plasmid analysis

**Figure 2:**
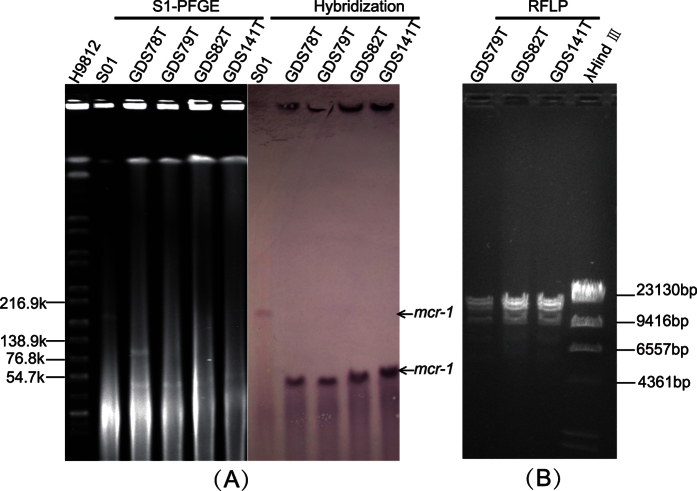
(**A**) Pulsed field gels of *S1* digested genomic DNA and Southern blot in gel hybridization with probe *mcr-1*. (**B**) ApaLI restriction digestion profiles of *mcr-1*-carrying plasmids of the three transconjugants.

**Figure 3:**
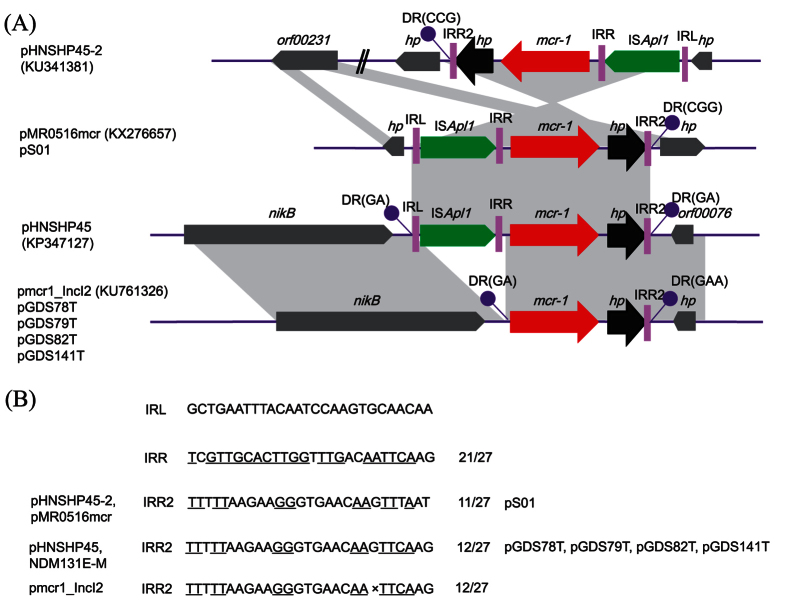
(**A**) The genetic context surrounding the *mcr-1* gene and structural comparison with plasmids pHNSHP45-2 (KU341381), pMR0516mcr (KX276657), pHNSHP45 (KP347127) and pmcr1_IncI2 (KU761326). The arrows indicate the positions and directions of transcription for each gene. Regions of >99% homology are marked by grey shading. The names of plasmids in this study are highlighted in bold. DR, direct repeats; IRL, terminal inverted repeats at the left; IRR, terminal inverted repeats at the right. (**B**) Sequence feature of the insertion site.

PCR-based replicon typing (PBRT) including IncHI2, IncI2, IncFIB and IncFII were detected in the original strains. While, only IncI2 was found in the three transconjugants. For GDS78T, both IncI2 and IncFII were identified (Table 1). *S1*-PFGE and Southern blot showed that *mcr-1* in all the four transconjugants was located in an IncI2 plasmid with size of 70 kb, approximately. In strain S01, the *mcr-1* was located in a round 190 kb IncHI2 plasmid (Fig. 2A). Restriction Fragment Length Polymorphism (RFLP) indicated that pattern of GDS79T was different from GDS82T and GDS141T (Fig. 2B). After 14 days of passage without colistin, the plasmids carrying *mcr-1* were stable both in the parent strains and transconjugants. PCR-mapping and sequencing showed that an approximately 2,600 bp long fragment designed as the *mcr-1* cassette^26^, was inserted between *nikB* and *ydfA* on the backbone of IncI2 plasmid in the four transconjugants. The *mcr-1* cassette encompasses the likely promoter sequences, the *mcr-1* gene and a hypothetical protein^26^. Nevertheless, compared with pHNSHP45 (KP347127), the firstly identified *mcr-1*-carrying IncI2 plasmid, the IS*Apl1* is absent from the upstream of the *mcr-1* gene (Table 2). In the original strain S01, we then confirmed that a 3,679 bp length of *ISApl1-mcr-1* fragment was inserted in approximately 8,500 bp downstream of *terY2* on pHNSHP45-2 (KU341381), which is in accordance with pMR0516mcr (KX276657) (Fig. 3).

### Molecular typing

**Figure 4:**
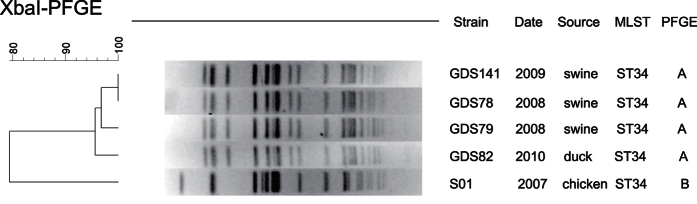
Pulsed-field gel electrophoresis fingerprinting patterns of *XbaI*-digested total DNA preparations from *Salmonella* strains harbouring *mcr-1*.

All of the five isolates were successfully typed by pulsed-field gel electrophoresis (PFGE), and two different PFGE clusters designated A and B were obtained (Fig. 4). Cluster A contains four isolates, three of which were isolated from different pig farms in 2008 and 2009, while the remaining one was isolated from duck in 2010. The single strain of cluster B was isolated from chicken in 2007. Multi-locus sequence typing showed that the five *mcr-1*-positive *S*. Typhimurium belongs to ST34, though they were classified into two PFGE clusters (Fig. 4).

## Discussion

Since initial identification in China, the *mcr-1* gene has been detected in *Enterobacteria* from almost 30 countries on five continents^16^. The *mcr-1* gene was frequently detected in *Enterobacteriaceae*, including *E. coli*^15^,^27^,^28^, *Enterobacter aerogenes* and *Enterobacter cloacae*^18^, *Klebsiella pneumonia*^15^, *Shigella somnei*^29^, as well as *Salmonella enterica*^30^. Among them, *Salmonella enterica* has attracted much attention owing to it as an important food-borne pathogen. *Salmonella enterica* harbours a number of serovars, but the *mcr-1* is not restricted to a certain serovar, which has been identified in *Salmonella enterica* serotype Anatum, Derby, 1,4,[5],12:i:2,31 Java, Paratyphi B, Rissen, Schwartzengrund, Typhimurium and Virchow^16^. Moreover, several reports confirmed the *mcr-1*-positive *Salmonella enterica* were multidrug resistance strains, which usually carry other resistance genes including ESBL genes and quinolone resistance genes^25^,^31^,^32^. More recently, the *mcr-1* gene was found in the multidrug resistant and copper-tolerant *Salmonella spp.* from pigs^33^. Notably, the *mcr-1*-positive *Salmonella* isolates were strongly associated with the particular successful MDR clonal lineages, including *S.* 1,4,[5],12:i:/ST34 and *S.* Rissen/ST469, which had widely spread epidemically in European countries^33^. Recently, *mcr-1* was detected in MDR *S.* Typhimurium/ST34 and *S.* Typhimurium/ST36 in England and Wales^32^. Here, all the five *mcr-1*-positive *Salmonella* strains belong to *S.* Typhimurium/ST34, which is the predominant ST of *S.* Typhimurium in Guangdong, China^34^. In our previous study, we have characterized a high prevalence of MDR *S.* Typhimurium/ST34 carrying PMQR^8^, which was also frequently detected in human clinical and food samples in Hong Kong in the same period^8^. The dissemination of this clone carrying *mcr-1* from food-producing animals to humans via food chain might expedite colistin resistance in *Salmonella* strains.

The *mcr-1* gene was so far associated with diverse plasmids belonging to the IncI2, IncHI2, IncP, IncX4, IncFII and IncF replicon types^15^,^35^,^36^,^37^,^38^,^39^. Among them, IncHI2 and IncX4 rather than IncI2 plasmids, which were firstly identified to harbour the *mcr-1* gene in *E. coli*, were the most common replicons in *Salmonella spp*^10^,^32^,^36^. However, the *mcr-1* gene was located on the IncI2 plasmid in all *S.* Typhimurium/ST34 strains except strain S01 in this study, which indicated that the IncI2 plasmid harbouring the *mcr-1* gene has circulated between *E. coli* and *Salmonella spp*. Interestingly, the *mcr-1*-positive IncI2 plasmid was co-occurrence with the other plasmids with diverse replicon types such as IncHI2 and IncFII in the four clonal *mcr-1*-positive strains. Unlike the original *E. coli* SHP45, which carried two *mcr-1*-positive plasmids including an IncI2 type pHNSHP45 and an IncHI2 type pHNSHP45-2^15^,^40^, the *mcr-1* gene was only found on IncI2 plasmid in this study. This indicated that other mobile elements were probably involved in the mobilization of the *mcr-1* gene, in addition to plasmids. Indeed, IS*Apl1* was present upstream of the *mcr-1* gene on both *mcr-1*-carrying plasmids in *E. coli* SHP45, while it was absent on all the *mcr-1* positive IncI2 plasmids in this study.

Only five *mcr-1*-positive strains were identified in 276 isolates, indicating that the *mcr-1*-positive *Salmonella* isolates are sporadic in animals in China. It is possible that the transferable *mcr-1* gene here appears in the form lacking the insertion sequence IS*Apl1*^41^. In addition, all the *mcr-1*-positive *Salmonella enterica* were isolated from the disease animals, whereas most of the *mcr-1*-negative colistin resistant strains were from healthy animals. Although the prescription was not recorded during the sampling, existing evidence suggested that exposure to colistin was closely related to its resistance rates^14^. The high rate of colistin resistance and low *mcr-1* positive rates showed that the plasmid-mediated colistin resistance was not the main way conferred colistin resistance among *Salmonella* isolates. It is of possibility that other colistin-resistant mechanisms or even novel *mcr-1* type exisit^35^, which needs to be evaluated in future study.

In conclusion, we reported five *mcr-1*-positive *Salmonella* isolates from animals in China between 2007 and 2015. Clonal spread of PMQR-carrying ST34 *Salmonella* isolates and horizontal transmission of IncI2 plasmids were the main way to disseminate *mcr-1* gene in this study. Colistin should be used more prudently in food-producing animals to prevent *mcr-1* gene spreading between different specials.

## Materials and Methods

### Bacterial strains

A total of 276 nonduplicate *Salmonella enterica* isolates (246 from avian and 30 from swine), isolated from faecal swabs of healthy or sick animals at poultry farms, swine farms and two diagnostic laboratories in Guangdong and Shandong province in China among 2007 and 2015, were used in this study (Table 3). Among them 127 were *S.* Typhimurium, 79 were *Salmonella enterica* serotype Indiana, six were *Salmonella enterica* serotype Enteritidis, two were *Salmonella enterica* serotype Meleagridis, two were *Salmonella enterica* serotype Bredeney, one was *Salmonella enterica* serotype Abaetetuba and 59 were non-typeble. The details of these strains have been described in our previous works^7^,^42^.

### Screening of the *mcr-1* gene and susceptibility testing

MICs of Colistin were determined by the agar dilution method according to the guidelines of the Clinical and Laboratory Standards Institution^43^,^44^. Colistin-resistant isolates were screened for the presence of *mcr-1* by PCR with the primers as described by Liu *et al*.^15^. All the *mcr-1*-positive isolates were detected by other resistance determinants including PMQR genes, *rmtB, armA* and *floR*. Susceptibility testing was also assessed in the *mcr-1*-positive isolates using the following antimicrobial agents: ampicillin (AMP), cefotaxime (CTX), cefoxitin (FOX), meropenem (MEM), streptomycin (STR), amikacin (AMK), gentamicin (GEN), florfenicol (FFC), tetracycline (TET), nalidixic acid (NAL), ciprofloxacin (CIP), olaquindox (OQX), trimethoprim-sulfamethox (SMZ/TMP), and fosfomycin (FOS). *E. coli* ATCC 25922 was used as a quality control strain.

### Conjugation and transformation analysis

*E. coli* C600 was used as the recipient for the conjugation experiment of MCR-producing *Salmonella* isolates. The transconjugants were selected on MacConkey agar containing colistin (2 mg/L) and streptomycin (2,000 mg/L), and finally confirmed by PCR and ERIC-PCR^45^. Plasmids that are not transferable by conjugation were studied by transformation assay. Plasmid DNA was extracted using a QIAGEN Prep Plasmid Midi Kit. Purified plasmids were used in electroporation experiments with *E. coli* DH5α following the manufacturer’s instructions. Transformants were incubated at 37 °C for 1 h and were then selected on LB agar containing 2 mg/L colistin.

### Plasmid Characterization

Incompatibility (Inc) groups were assigned by PBRT. To analyze the location of the *mcr-1* gene, *S1* nuclease-PFGE and Southern blot analysis were performed. Briefly, whole-cell DNA of the donor strains and the transconjugants harbouring *mcr-1* were extracted and embedded in agarose gel plugs. Subsequently, the agarose gel plugs were treated with *S1* nuclease (TaKaRa, Dalian, China) and the DNA fragments were separated by PFGE. Southern blot hybridization was then performed with DNA probes specific for the *mcr-1* gene, which was non-radioactively labeled with a DIG High Prime DNA labeling and detection kit (Roche Diagnostics, Mannheim, Germany). Transconjugants containing one plasmid were extracted and analyzed by RFLP using *ApaLI* (TaKaRa, Dalian, China) digestion. The genetic context surrounding the *mcr-1* gene was investigated by PCR mapping and sequencing according to the plasmids sequences which had been submitted to GenBank. The primers used to determine the regions upstream and downstream of the *mcr-1* gene are listed in Table 2. To assess the stability of *mcr-1*-positive plasmids, the original strains and transconjugants were cultured by in daily serial passages with the absence of colistin for two weeks.

### Molecular typing

Genomic DNA of the *mcr-1*-positive isolates was analyzed by PFGE following digestion with *XbaI*^46^. *Salmonella enterica* serotype Braenderup H9812 standard was used as size marker. Comparison of PFGE patterns was performed by BioNumerics®v6.6 (Applied Maths, Ghent, Belgium) with a cut-off at 90% of the similarity values to indicate identical PFGE types. Multi-locus sequence typing (MLST) was performed by using the primers and protocol specified at the *Salmonella enterica* MLST website (http://mlst.warwick.ac.uk/mlst/dbs/Senterica).

## Additional Information

**How to cite this article**: Li, X.-P. *et al*. Clonal spread of *mcr-1* in PMQR-carrying ST34 *Salmonella* isolates from animals in China. *Sci. Rep.* **6**, 38511; doi: 10.1038/srep38511 (2016).

**Publisher's note:** Springer Nature remains neutral with regard to jurisdictional claims in published maps and institutional affiliations.

## Figures and Tables

**Figure 1:**
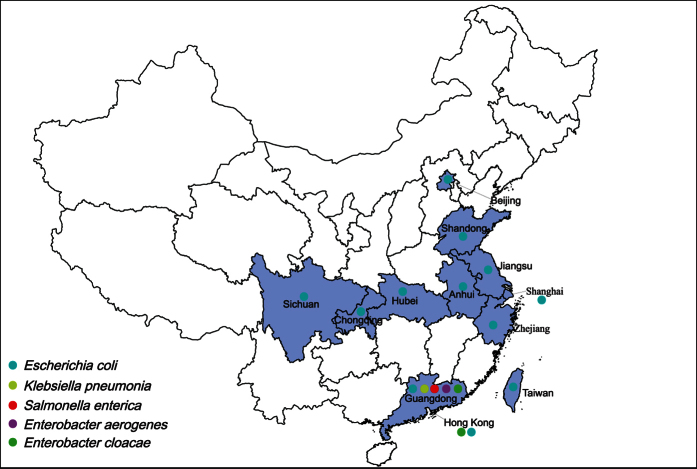
Distribution of the *mcr-1* colistin resistance gene in China by 30 Aug, 2016. (Created by ArcGIS 10.3 software, ESRI Inc., Redlands, CA, USA, available at: https://www.arcgis.com/home/).

**Table 1:** Characteristics of the five *Salmonella enterica* isolates carrying *mcr-1*.

Strain^a^	Colistin MIC	Other resistant profile^b^	Resistance gene(s)^b^	Plasmid	
				Replicon type^b^	Size of plasmid harbouring *mcr-1* (kb)
S01	16	OLA, CIP, NAL, AMP, STR, GEN, FFC, TET, SMZ/TMP	*mcr-1, oqxAB, aac*(*6*′)*-Ib-cr, floR*	HI2	～190
GDS78	16	OLA, NAL, AMP, STR, GEN, FFC, TET, SMZ/TMP	*mcr-1, aac*(*6*′)*-Ib-cr*	HI2, I2, FIB, FII	～70
GDS79	16	OLA, NAL, AMP, STR, GEN, FFC, TET, SMZ/TMP	*mcr-1, oqxAB, aac*(*6*′)*-Ib-cr, floR*	HI2, I2, FIB, FII	～70
GDS82	16	OLA, NAL, AMP, STR, GEN, FFC, TET, SMZ/TMP	*mcr-1, oqxAB, aac*(*6*′)*-Ib-cr, floR*	HI2, I2, FIB, FII	～70
GDS141	16	OLA, NAL, AMP, STR, GEN, FFC, TET, SMZ/TMP	*mcr-1, oqxAB, aac*(*6*′)*-Ib-cr, floR*	HI2, I2, FIB, FII	～70

^a^Isolates from which the *mcr-1* gene can be transferred to the recipient by conjugation are underlined.

^b^AMP, cefotaxime; STR, streptomycin; GEN, gentamicin; FFC, florfenicol; OLA, olaquindox; CIP, ciprofloxacin; NAL, nalidixic acid; TET, tetracycline; SMZ/TMP, trimethoprim/sulfamethoxazole. The antimicrobial susceptibility results were interpreted according to breakpoint of CLSI (M100-S25), except that florfenicol (≥32 μg/mL) was interpreted according to breakpoint of veterinary CLSI (VET01-A4/VET01-S3). All isolates were susceptible to amikacin, cefotaxime, cefoxitin, fosfomycin, and meropenem. Resistance phenotypes, genes and plasmids transferred to the recipient by conjugation are underlined.

**Table 2:** Primers used in PCR mapping of the genetic context surrounding the *mcr-1* gene in the *Salmonella* isolates from animals in China.

PCR Primer^a^	Primer sequence (5′→3′)	Target DNA sequence	Position	PCR amplification in this study^b^					Reference sequence
				S01	GDS78	GDS79	GDS82	GDS141	
ISApl1-F	AGAATTTTACTTCCCCGAGCC	IS*Apl1*	21995..22015	+	−	−	−	−	pHNSHP45 (KP347127)
M-1R	CGCGCCCATGATTAATAGCAA	*mcr-1*	22656..22676						
nikB-F	GATTATTTTACGCCCGGAGCA	*nikB*	19093..19113	ND	+	+	+	+	pHNSHP45 (KP347127)
M-2R	AGCAAGGCATTGTCATAAGCA	*mcr-1*	23699..23719						
I-F	CAAAGACGCGGTACAAGCAAC	*mcr-1*	23081..23101	ND	+	+	+	+	pHNSHP45 (KP347127)
I-R	AAGACTCGTCCAACATATGGC	*orf076*	24907..24927						
H-1F	AACGCTTCGAAATAACGGAT	orf00200	151220..151239	−	ND	ND	ND	ND	pHNSHP45-2 (KU341381)
H-1R	GTTGTATTTATTACCGATGGC	terF	155812..155832						
H-2F	ACCATGGACGTGAAAATGCT	—	50090..50109	+	ND	ND	ND	ND	pMR0516mcr (KX276657)
H-2R	AGATTAGAGAGTGCCCCTCCC	—	45927..45947						

^a^F, forward primer; R, reverse primer.

^b^ND, not detected.

**Table 3:** Colistin resistant and *mcr-1* positive *Salmonella* strains from animals in China, 2007–2015.

Year	Animals	Strains tested for colistin MIC	Resistant to colistin	Proportion of *mcr-1* positive (n) among colistin-resistant *Salmonella* strains (N)^a^
2007	Avian	44	2	1/2
2008	Avian	53	5	0/5
2008	Swine	7	3	2/3
2009	Avian	28	1	0/1
2009	Swine	3	1	1/1
2010	Avian	11	4	1/4
2010	Swine	3	0	N.a.
2012	Avian	42	5	0/5
2014	Avian	17	0	N.a.
2014	Swine	11	0	N.a.
2015	Avian	51	1	0/1
2015	Swine	6	0	N.a.

^a^N.a. means not available.
